# Associations between postmigration living situation and symptoms of common mental disorders in adult refugees in Europe: updating systematic review from 2015 onwards

**DOI:** 10.1186/s12889-023-15931-1

**Published:** 2023-07-05

**Authors:** Anna Christina Nowak, Niklas Nutsch, Tessa-Maria Brake, Lea-Marie Gehrlein, Oliver Razum

**Affiliations:** 1 Department of Epidemiology and International Public Health, School of Public Health, Bielefeld University, Bielefeld, Germany; 2 Department of Population Medicine and Health Services Research, School of Public Health, Bielefeld University, Bielefeld, Germany

**Keywords:** Asylum seekers, Adult refugees, Postmigration living difficulties, Postmigration stressors, Mental health, European countries, Social determinants of health

## Abstract

**Background:**

Refugees and asylum seekers have a high prevalence of psychiatric disorders such as post-traumatic stress disorder (PTSD), anxiety, and depression. The postmigration context inheres different risk and protective factors for mental health of refugees and asylum seekers in host countries. We conducted a systematic review to update knowledge on the association between characteristics of the postmigration living situation (PMLS) and mental health outcomes in Europe since 2015.

**Methods:**

We searched in five databases according to the PRISMA statement. From a total of 5,579 relevant studies published in 2015–22, 3,839 were included for title and abstract screening, and 70 full texts screened for eligibility. Out of these, 19 studies on refugees and asylum seekers conducted in European countries after 2014 were included in this systematic review. The quality of studies was assessed by using the Mixed Methods Appraisal Tool (MMAT) – version 2018. We performed a narrative synthesis using the four layers of the social determinants of health framework.

**Results:**

A wide range of risk and protective factors for mental health in the PMLS were identified as exposure measures, which included individual factors (e.g., language skills), social and community networks (e.g., family concerns, loneliness and social support, discrimination), living and working conditions (e.g., legal status, duration of residence, unemployment and financial hardship, housing) as well as general socio-economic, cultural and environmental factors (e.g., social status, acculturation). We found postmigration stressors are positively associated with symptoms of depression, anxiety, and PTSD, albeit not consistently so. Especially, the general socio-economic, cultural and environmental factors showed weak associations with mental health.

**Conclusions:**

Heterogenous study characteristics likely explain the inconsistent associations between characteristics of the PMLS and mental health outcomes. However, broken down in its component layers, most risk and protective factors of the PMLS were significantly associated with symptoms of mental disorders showing the same direction of association across the included studies, while the association between some stressors or resources of the PMLS and mental health turns out to be less homogeneous than expected. Characteristics of the PMLS contribute to the high prevalence of mental diseases of refugees and asylum seekers. Disadvantages in general socio-economic conditions, living and working conditions, in access to social and community networks need to be redressed, in addition to better access to health care.

**Supplementary Information:**

The online version contains supplementary material available at 10.1186/s12889-023-15931-1.

## Introduction

Between 2014 and 2018, more than 4 million persons sought asylum in one of the member states of the European Union, with a peak in 2015 and 2016. Thereafter, the number of new arrivals declined until Russia’s war in Ukraine in 2022 [1]. The increase in the number of asylum seekers in 2015 and 2016 posed challenges to the social and health care systems of European countries. Mental health systems did not have the capacity to diagnose and treat all those in need [2], even though refugees and asylum seekers are at high risk of mental disorders, particularly post-traumatic stress disorder (PTSD), depression, and anxiety. According to the ICD-10 classification [3], PTSD is defined as a delayed mental reaction to a stressful, threatening event. Depression is characterized by low mood, decreased energy and activity, whereas anxiety disorders are defined as disorders that occur predominantly in well-defined, currently non-hazardous situations; the patient's apprehension may be manifested by, e.g., palpitations and is often associated with secondary anxieties such as loss of control [3]. Refugee and asylum seeker populations have prevalences of 4.4–86.0% for PTSD, 2.3–80% for depression, and 20.3–88.0% for anxiety [4]. Differences in prevalence estimates are explained by differences in study population, study quality, legal status, length of stay, and differences between host societies [4].

The likelihood of developing depression, PTSD or anxiety disorder significantly increases with the number of traumatic events experienced [4–6]. A meta-analysis by Hou et al. (2020) [7] found that postmigration stressors regarding interpersonal interactions and combined subjective, interpersonal and material stressors were associated with anxiety, depression and PTSD. The postmigration living situation (PMLS) in host countries include all kinds of social determinants of health that might constitute risk factors (stressors or living difficulties) or protective factors (resources) for symptoms of mental disorders. The WHO defined social determinants of health (SDH) as “non-medical factors that influence health outcomes” and “the conditions in which people are born, grow, live, work and age” [8]. In the framework, social determinants are formulated neutrally, but can have both positive and negative effects on health. In this paper, we use the term postmigration living difficulty (PMLD) to describe stressful life events and the term postmigration living situation (PMLS) as a more neutral formulation that can include both positive and negative aspects of the living situation of refugees and asylum seekers in European host countries. This social determinants of health framework is used to describe the marginalized living situation of refugees and asylum seekers [9]. Davies et al. [10] draw attention to the importance of migration for the different layers in the SDH-Framework and at the same time emphasize the implications of the layers for the health of migrants. For example, Hynie [9] describes income, employment, housing, language skills, the asylum-seeking process, social support and isolation and discrimination as relevant social determinants that result from policies, societal and interpersonal attitudes and living environments.

To broaden the understanding of the PMLS of refugees and asylum seekers, we performed an updated systematic review (2015–2022) examining the association between characteristics of the postmigration living situation and symptoms of PTSD, anxiety and/or depression among recently arrived adult refugees in European countries.

## Methods

We conducted a systematic review according to the PRISMA Statement [11]. A review protocol was registered in PROSPERO (ID: CRD42022320601). We made changes compared to the review protocol during the data extraction process: we extended our understanding from PMLD to PMLS and changed the risk of bias quality assessment because of better suitability of the heterogenous study designs.

We searched four public health and psychology databases (PubMed, Web of Science, PsycINFO and Psyndex) in February 2022, complemented by a search in Google Scholar in October 2022 to include grey literature.

The research question was constructed using the PICO strategy (s. Table 1). The search strategy comprises a combination of free text search terms and subject headings (MeSH) related to three concepts: 1) asylum seekers and refugees, 2) characteristics of the postmigration living situation (PMLS) as exposure measures, considering specific factors according to the model of the social determinants of health [12], and 3) mental health and disorder, including PTSD, anxiety, and depression as outcome parameters. Search terms were connected using Boolean operators. Search strategies were slightly modified for each database (see Additional file 1). Additionally, we manually searched reference lists of included studies and reviewed articles for relevant publications.

**Table 1:** Elements of the research question (PICO)

PICO	Definition
Population	Adult asylum seekers and refugees recently arrived in European countries
Intervention / Exposure	Characteristics of the postmigration living situation (PMLS) that might constitute risk factors (stressors or living difficulties) or protective factors (resources) for mental health
Comparison	Not applied
Outcome	Symptoms of PTSD, anxiety, and/or depression

The eligibility criteria for inclusion or exclusion of primary studies identified in the search were defined based on the research question and the PICO-model. In this regard, studies were only included in the systematic review if the population studied were adult refugees or asylum seekers living in European countries. Studies on children and adolescents were thus excluded as evidence suggests that this population subgroups face different special stressors and resources in the postmigration context after their flight [13]. Regarding the exposure, the measurement of characteristics of the PMLS had to be described clearly, focusing one of the social determinants included in the model by Whitehead and Dahlgren [12]. Only published studies investigating the association between risk factors (stressors or living difficulties) or protective factors (resources) of the PMLS and symptoms of PTSD, depression and anxiety were considered. The mental health outcomes had to be assessed by using validated scales or checklists according to ICD-10, DSM IV or DSM V. We only considered PTSD, depression, and anxiety as outcomes because they are widely studied in refugees and asylum seekers [14], whereas studies on other psychological disorders are less researched or of poor quality [15]. Studies assessing psychological distress were excluded as it was not possible to assign this outcome to the outcomes of interest on symptoms of PTSD, depression, or anxiety.

We included primary studies with quantitative data, such as cross-sectional, longitudinal, cohort and mixed-methods studies. Only studies published in German or English language were considered.

Due to the significant increase in the number of refugees and asylum seekers in the European Union from 2015 onwards [1], the accompanying media and political attention and the particular legal, social and care challenges at this time, we only included studies that were published from 2015 onwards with data collection conducted between 2014 and 2022. We chose the time of data collection because of the limited accuracy of the durations of stay of refugees and asylum seekers in the included primary studies. This means that population groups who arrived before 2014 were also included if the data collection took place between 2014 and 2022 because they were also affected by the challenges of political, social and health systems during this time. Studies that did not provide information on the data collection date were excluded. Our aim was to determine whether the significance of the post-migration living situation on mental health has changed in recently arrived populations and provide a literature update. Studies from European countries were considered, as there should be comparable standards for the treatment of asylum seekers and refugees in the receiving countries through the establishment of a Common European Asylum System. For example, the Reception Directive [16] regulates living conditions in terms of accommodation, family reunification, access to education for children, access to the labor market or health care during the asylum procedure.

In the first step of the literature screening process, two reviewers independently screened the publications by title and abstract and assessed them against the eligibility criteria. In the second step, two reviewers independently screened the full texts. Disagreements were discussed within the reviewer team. The interrater reliability for full text screening was calculated and revealed a substantial agreement level with a Kappa value of 0.72 (SD: 0.12) and with an agreement of 87.14% [17]. We screened articles using Rayyan, a software for collaborative systematic reviews [18].

Relevant data were extracted using an extraction form developed by the lead author (ACN). Data extraction was performed by four reviewers in Microsoft Excel. Although data extraction was not conducted independently, extracted data were discussed within the team. Narrative synthesis was conducted to cluster and compare study results according to the model of the social determinants of health [12], which includes individual lifestyle factors (layer 1), social and community networks (layer 2), living and working conditions (layer 3) and general socio-economic, cultural and environmental conditions (layer 4). No meta-analysis was performed because of heterogeneity of included studies. For better comparability, where applicable only results of adjusted models will be reported.

For critical appraisal of studies, we used the Mixed Methods Appraisal Tool (MMAT) – version 2018 because it allows critical quality appraisal of qualitative, quantitative and mixed-methods studies based on five core quality criteria [19]. It has a high to moderate interrater reliability [20]. The assessment was conducted by two authors independently. Disagreements were resolved within the publication group to minimize the risk of bias across studies. To ensure high internal validity, low-quality studies were excluded.

## Results

**Fig. 1:**
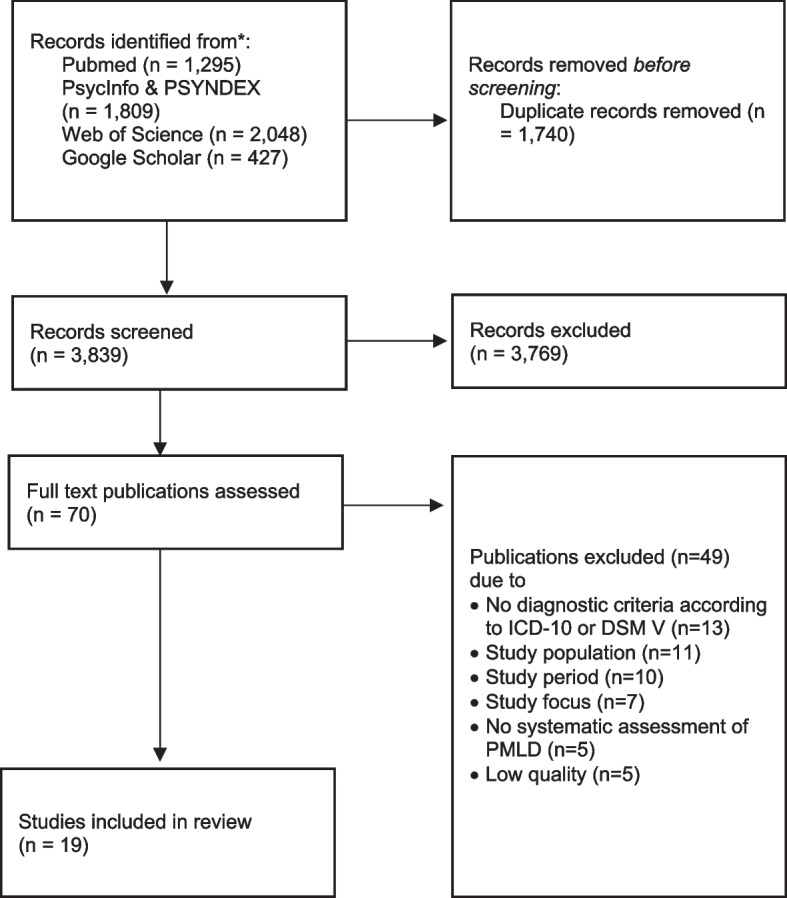
Prisma flow diagram showing the selection of studies

We screened 3,839 records after removing duplicates. Figure 1 shows the PRISMA flow diagram, indicating how we selected studies for inclusion or exclusion.

We included *n* = 19 studies conducted in Germany (*n* = 9), Sweden (*n* = 4), Italy (*n* = 1), Switzerland (*n* = 1), Norway (*n* = 2), the Netherlands (*n* = 1), and Austria (*n* = 1). 14 (73.7%) of the studies had a cross-sectional, three (15.8%) a longitudinal study design, and one study each a mixed-methods and a RCT design. The number of participants ranged from 57 to 4,325. Half of the studies included a diverse study population (*n* = 9), eight studies (40%) included refugees from Syria, one study refugees from Afghanistan and Iraq, one study refugees from Afghanistan only. For more information on study characteristics, see Table 2.

**Table 2:** Overview of the included studies

Author(s)(Year)	Country	Study population / Year(s) of data collection	Study design	Risk and protective factors of the PMLS	Symptoms			Size of association	Outcome measure	Co-/Variables	Study quality (MMAT)
					**Depression**	**Anxiety**	**PTSD**				
Barbieri et al. (2021) [21]	Italy	African refugees and Asylum Seekers (*n* = 122) 2016–2019	Cross-sectional study	**Legal status** *(asylum seeker vs. visa [Ref])*	-			-	PCL-5	**Sociodemographic factors** (age, gender, education); **Post-migration factors** (legal status, duration of residence, employment, accommodation); **Trauma-related factors** (number of trauma types)	High
						-		-			
							⇅	OR_3vs1_ = 1.24 [CI 0.12, 13.02] OR_3vs2_ = 9.44 [CI 0.66, 134.93] OR_2vs1_ = 0.13 [CI 0.01, 2.16]			
				**Duration of residence** *(months since residing in host country)*	-			-			
						-		-			
							⇅	OR_3vs1_ = 0.97 [CI 0.91, 1.04] OR_3vs2_ = 0.95 [CI 0.90, 1.00] OR_2vs1_ = 1.03 [CI 0.97, 1.08]			
				**Employment** *(unemployed vs. employed [Ref])*	-			-			
						-		-			
							⇅	OR_3vs1_ = 1.59 [CI 0.48, 5.21] OR_3vs2_ = 2.19 [CI 0.77, 6.24] OR_2vs1_ = 0.73 [CI 0.23, 2.26]			
				**Accommodation** *(large-reception centers with* > *1000 people vs. small-medium reception centers with* < *1000 people [Ref])*	-			-			
						-		-			
							↑	**OR**_**3vs1**_** = 12.77 [CI 1.49, 109.44]** **OR**_**3vs2**_** = 6.68 [CI 1.81, 24.61]** OR_2vs1_ = 1.91 [CI 0.19, 18.81]			
Böge et al. (2020) [22]	Germany / Jordan	Syrian refugees and asylum seekers (*n* = 89) 2017–2018	Cross-sectional study	**Social support** *(MSPSS)*	↓			**β = -0.240 [CI NA]**	PHQ-9 GAD-7 HTQ	-	Moderate
						↓		β = -0.042 [CI NA]			
							↓	**β = -0.230 [CI NA]**			
Borho et al. (2020) [23]	Germany	Syrian refugees (T_1_: *n* = 200, T_2_: *n* = 108) T1: 2017; T2: 2019	Two-wave longitudinal study	**Perceived discrimination**	↑			**β**_**T1**_** = 0.235 [CI 0.219, 3.552]** **β**_**T2**_** = 0.271 [CI 0.383, 2.769]**	PHQ-9 GAD-7 ETI	**Sociodemographic factors** (age, gender, education); **Trauma-related factors** (number of traumatic events); **Post-migration factors** (accommodation, employment, duration of residence, future validity of permit, discrimination)	High
						↑		**β**_**T1**_** = 0.263 [CI 0.359, 2.988]** **β**_**T2**_** = 0.335 [CI 0.700, 2.738]**			
							↑	N.A			
				**Future validity of permit** *(in months)*	-			N.A			
						-		N.A			
							↓	N.A **β**_**T2 **_**= -0.184 [CI -0.388, -0.035]**			
				**Duration of residence** *(months since residing in host country)*	-			N.A			
						-		N.A			
							-	N.A			
				**Accommodation** (*Collective accommodation center, Own apartment alone or with family, Shared flat)*	-			N.A			
						-		N.A			
							-	N.A			
Costa et al. (2020) [24]	Germany	Diverse refugee sample (*n* = 560) 2018	Cross-sectional study	**Subjective social status** *(3 or more steps down vs. no change [Ref])*	↑			**B = 1.048 [CI NA]**	PHQ-2 GAD-2	**Sociodemographic factors** (sex, age, education); **Post-migration factors** (SSS mobility, SSS in country of origin, duration of residence)	Moderate
						↑		**B = 1.006 [CI NA]**			
							-	-			
				**Subjective social status** *(1 or 2 steps down vs. no change [Ref])*	↓			B = -0.176 [CI NA]			
						↑		B = 0.114 [CI NA]			
							-	-			
				**Subjective social status** *(1 or 2 steps up vs. no change [Ref])*	↓			B = -0.499 [CI NA]			
						↓		B = -0.985 [CI NA]			
							-	-			
				**Subjective social status** *(3 or more steps up vs. no change [Ref])*	↑			B = 0.19 [CI NA]			
						↑		B = 0.269 [CI NA]			
							-	-			
Georgiadou et al. (2018) [25]	Germany	Syrian refugees (*n* = 200) 2017	Cross-sectional study	**Future validity of permit** *(in months)*	↑			β = 0.12 [CI -0.04, 0.24]	PHQ-9 GAD-7 ETI	**Sociodemographic factors** (sex, age, education, illiterate, marital status); **Post-migration factors** (accommodation, duration of residence, duration of residence permit, future validity of permit); **Pre-/Peri-migration factors** (escape journey, escape duration); **Trauma-related factors** (number of traumatic events); **Health-related factors** (PHQ-9 score, GAD score, ETI score, mental health treatment)	Moderate
						↑		β = 0.05 [CI -0.07, 0.13]			
							↓	**β = -0.20 [CI -0.58, -0.01]**			
				**Duration of residence permit** *(in months)*	↓			β = -0.15 [CI -0.22, 0.03]			
						↓		β = -0.08 [CI -0.13, 0.05]			
							↑	β = 0.19 [CI -0.04, 0.46]			
				**Duration of residence** *(in months)*	↓			β = -0.03 [CI -0.16, 0.11]			
						↑		β = 0.08 [CI -0.04, 0.15]			
							↓	β = -0.09 [CI -0.43, 0.11]			
				**Accommodation** *(own apartment / with family vs. collective accommodation center / with others [Ref])*	↑			β = 0.03 [CI -0.95, 1.70]			
						↓		β = -0.01 [CI -1.04, 0.82]			
							↑	β = 0.01 [CI -2.40, 2.89]			
Groen et al. (2019) [26]	Netherlands	Afghan and Iraqi refugees and asylum seekers (*n* = 57) 2012–2015	Mixed-methods study	**Legal status** *(asylum vs. refugee [Ref])*	↑			β = 0.193 [CI -0.143, 0.560]	HSCL-25 HTQ	**Sociodemographic factors** (age, sex); **Post-migration factors** (PMLD, acculturation, legal status); **Trauma-related factors** (number of experienced traumas)	High
						↑					
							↑	β = 0.241 [CI -0.096, 0.628]			
				**Acculturation** *(CRM-BS)*	↓			β = -0.113 [CI -0.625, 0.239]			
						↓					
							↓	β = -0.112 [CI -0.642, 0.247]			
				**PMLD** *(PMLP-CL)*	↑			**β = 0.428 [CI 0.170, 0.710]**			
						↑					
							↑	**β = 0.396 [CI 0.140, 0.713]**			
Gühne et al. (2021) [27]	Germany	Syrian refugees (*n* = 133) 2018–2019	Cross-sectional study	**Social support** *(ESSI)*	↓			N.A	PHQ-9 PDS-5	**Sociodemographic factors** (age, sex, number of household members); **Post-migration factors** (employment, social support, duration of residence)	High
						-		-			
							↓	N.A			
				**Duration of residence** *(months since residing in host country)*	↑			β = 0.016 [CI NA]			
						-		-			
							↑	β = 0.050 [CI NA]			
				**Employment** *(employed vs. unemployed [Ref])*	↓			**β = -2.506 [CI NA]**			
						-		-			
							↓	**β = -4.871 [CI NA]**			
Hecker et al. (2018) [28]	Switzerland	Diverse refugee sample (*n* = 94) 2015–2016	Cross-sectional study	**Social support** (SPS)	-			-	ITQ	**Sociodemographic factors** (gender); **Trauma-related factors** (trauma exposure); **Post-migration factors** (social support, PMLD)	Moderate
						-		-			
							↑	β = 0.13 [CI NA] **β = 0.22 [CI NA]** (for DSO)			
				**PMLD** *(PMLD-CL)*	-			-			
						-		-			
							↑	β = 0.17 [CI NA] **β = 0.42 [CI NA]** (for DSO)			
Kaltenbach et al. (2018) [29]	Germany	Diverse refugee sample (*n* = 57) 2015–2017	Longitudinal study	**Legal status**	-			N.A	PHQ-9 PSS-I PCL-5	-	High
						-		-			
							-	N.A			
				**Duration of residence**	-			N.A			
						-		-			
							-	N.A			
				**PMLD** *(PMLD-CL)*	-			N.A			
						-		-			
							↑	**N.A**			
Leiler et al. (2019) [30]	Sweden	Diverse refugee sample (*n* = 577) 2016–2017)	Cross-sectional study	**Legal status** *(asylum seekers vs. individuals with residence permit)*	-			N.A	PHQ-9 GAD-7 PC-PTSD	-	Moderate
						-		**N.A**			
							-	-			
Nissen et al. (2021) [31]	Norway	Syrian refugees (*n* = 902) 2018 -2019	Cross-sectional study	**Legal status** *(quota refugee vs. asylum seeker [Ref])*	↑			OR = 1.35 [CI 0.69, 2.65]	HSCL-25 HTQ	**Sociodemographic factors** (gender, age, education, marital status); **Post-migration factors** (legal status, duration of residence); **Migration-related factors** (arrived with friends/alone, length of flight, family members resettled in host country); **Trauma-related factors** (potentially traumatic experience)	Moderate
						↑		OR = 1.78 [CI 0.91, 3.49]			
							↑	OR = 1.20 [CI 0.60, 2.39]			
				**Legal status** *(family reunion vs. asylum seeker [Ref])*	↓			OR = 0.95 [CI 0.46, 1.98]			
						↑		OR = 1.28 [CI 0.62, 2.65]			
							↑	OR = 1.32 [CI 0.63, 2.79]			
				**Duration of residence** *(years since residing in host country)*	↑			**OR = 1.52 [CI 1.13, 2.05]**			
						↑		**OR = 1.52 [CI 1.14, 2.04]**			
							↑	OR = 1.30 [CI 0.96, 1.75]			
Nutsch & Bozorgmehr (2020) [32]	Germany	Diverse refugee sample (*n* = 4,136) 2016	Cross-sectional study	**Language skills** *(moderate vs. good [Ref])*	↑			OR = 1.144 [CI 0.841, 1.557]	PHQ-2	**Sociodemographic factors** (age, sex, marital status, education, nationality); **Post-migration factors** (legal status, asylum interview, employment, satisfaction with accommodation, language skills, loneliness); **Psychosocial factors** (self-esteem, resilient coping behavior, life satisfaction, anxiety)	High
						-		-			
							-	-			
				**Language skills** *(bad vs. good [Ref])*	↑			OR = 1.239 [CI 0,907, 1.692]			
						-		-			
							-	-			
				**Loneliness** *(LS-S)*	↑			**OR = 1.143 [CI 1.103, 1.184]**			
						-		-			
							-	-			
				**Legal status** *(rejected status vs. recognized status or asylum seekers [Ref])*	↑			**OR = 1.344 [CI 1.062, 1.701]**			
						-		-			
							-	-			
				**Asylum interview** *(yes vs. no [Ref])*	↓			**OR = 0.710 [CI 0.556, 0.908]**			
						-		-			
							-	-			
				**Employment** *(unemployed vs. employed [Ref])*	↑			**OR = 1.483 [CI 1.037, 2.121]**			
						-		-			
							-	-			
				**Satisfaction with accommodation**	↓			**OR = 0.943 [CI 0.909, 0.978]**			
						-		-			
							-	-			
Renner et al. (2021) [33]	Germany	Syrian refugees (*n* = 133) 2018–2019	Cross-sectional study	**Social support** *(ESSI)*	↓			**B = -0.32 [CI NA]**	PHQ-9 GAD-7 PDS-5	**Sociodemographic factors** (age, sex, education, living alone); **Post-migration factors** (employment, financial hardship, social support, connection to country of origin); **Trauma-related factors** (variability of traumatic events,); **Psychosocial factors** (self-efficacy, stigma, life satisfaction); **Other factors** (Religiousness)	High
						↓		**B = -0.20 [CI -0.39, 0.01]**			
							↓	**B = -0.55 [CI -0.98, -0.05]**			
				**Employment** *(employed vs. unemployed [Ref])*	↓			B = -1.51 [CI NA]			
						↓		B = -1.70 [CI -3.59, 0.17]			
							↓	B = -1.37 [CI -5.40, 2.93]			
				**Financial hardship** *(income* < *500€ vs. income* > *500€ [Ref])*	↑			B = 0.64 [CI NA]			
						↑		B = 0.88 [CI -1.34, 3.54]			
							↑	**B = 7.04 [CI 0.79, 13.72]**			
Schiess-Jokanovic et al. (2021) [34]	Austria	Afghan refugees (*n* = 93) 2019–2020	Randomized Controlled Trail (RCT)	**Language acquisition and barriers**	-			-	ITQ	-	Moderate
						-		-			
							-	**N.A**			
				**Family concerns**	-			-			
						-		-			
							-	N.A			
				**Perceived discrimination**	-			-			
						-		-			
							-	N.A			
				**Distress of asylum procedure**	-			-			
						-		-			
							-	N.A			
				**Socioeconomic living situation**	-			-			
						-		-			
							-	N.A			
Sengoelge et al. (2020) [35]	Sweden	Diverse refugee sample (*n* = 455) 2016–2018	Cross-sectional study	**Social and financial hardship** *(RPMS)*	↑			**B = 0.786 [CI 0.598, 1.021]**	HSCL-25	-	Moderate
						↑					
							-	-			
				**Social support** *(ESSI)*	↑			**B = -0.103 [CI NA]**			
						↑					
							-	-			
Solberg et al. (2020) [36]	Sweden	Diverse asylum-seeker sample (*n* = 455) 2016–2018	Cross-sectional study	**Language difficulties** *(yes vs. no [Ref])*	↑			**OR = 1.95 [CI 1.18, 3.23]**	HSCL-25 HTQ	**Sociodemographic factors** (gender, age, education, marital status); **Trauma-related factors** (potentially traumatic pre-/peri-migratory events); **Post-migration factors** (discrimination, language difficulties, financial hardship, missing social life back home, sadness due to lack of family reunification, social isolation, family conflicts)	Moderate
						↑		**OR = 2.02 [CI 1.26, 3.26]**			
							↑	**OR = 5.43 [CI 1.87, 5.18]**			
				**Missing social life back home** *(yes vs. no [Ref])*	↑			**OR = 2.09 [CI 1.23, 3.57]**			
						↑		**OR = 2.18 [CI 1.31, 3.62]**			
							↑	OR = 1.23 [CI 0.74, 2.04]			
				**Sadness due to lack of family reunification** *(yes vs. no [Ref])*	↑			OR = 1.14 [CI 0.65, 2.01]			
						↑		OR = 1.32 [CI 0.77, 2.27]			
							↓	OR = 0.76 [CI 0.43, 1.33]			
				**Family conflicts** *(yes vs. no [Ref])*	↑			**OR = 9.44 [CI 2.81, 31.72]**			
						↑		**OR = 4.72 [CI 2.09, 10.70]**			
							↑	**OR = 3.85 [CI 1.76, 8.42]**			
				**Social isolation** *(yes vs. no [Ref])*	↑			**OR = 3.10 [CI 1.76, 5.44]**			
						↑		**OR = 3.97 [CI 2.34, 6.71]**			
							↑	**OR = 5.69 [CI 3.25, 9.96]**			
				**Perceived discrimination** *(yes vs. no [Ref])*	↑			**OR = 4.26 [CI 1.43, 12.70]**			
						↑		**OR = 2.22 [CI 1.01, 4.90]**			
							↑	**OR = 5.43 [CI 2.00–14.75]**			
				**Financial hardship** *(yes vs. no [Ref])*	↑			**OR = 3.58 [CI 1.91, 6.72]**			
						↑		**OR = 2.95 [CI 1.70, 5.13]**			
							↑	**OR = 5.85 [CI 3.14, 10.89]**			
Strømme et al. (2021) [37]	Norway, Lebanon	Syrian refugees (*n* = 353) T0: 2017–2018 T1: 2018–2019	Longitudinal study	**Social support** *(poor vs. not poor [Ref])*	↑			**RR**_**T0**_** = 1.6 [CI 1.2, 2.1]** **RR**_**T1**_** = 6.2 [CI 3.6, 10.8]**	HSCL-10	**Sociodemographic factors** (age, gender) **Post-migration factors** (social support, economy);	High
						↑					
							-	-			
				**Economy** *(Poor vs. not poor [Ref])*	↑			RR_T0_ = 1.1 [CI 0.7, 1.7] **RR**_**T1**_** = 4.5 [CI 2.6, 7.9]**			
						↑					
							-	-			
Tinghög et al. (2017) [38]	Sweden	Syrian refugees (*n* = 1,215) 2016	Cross-sectional study	**Language difficulties** *(yes vs. no [Ref])*	↑			**OR = 2.39 [CI 1.78, 3.19]**	HSCL-25 HTQ	**Sociodemographic factors** (gender, age, education, marital status); **Trauma-related factors** (potentially traumatic pre-/peri-migratory events); **Post-migration factors** (discrimination, language difficulties, financial hardship, missing social life back home, sadness due to lack of family reunification, social isolation, family conflicts)	Moderate
						↑		**OR = 1.77 [CI 1.30, 2.40]**			
							↑	**OR = 2.77 [CI 2.00, 3.83]**			
				**Missing social life back home** *(yes vs. no [Ref])*	↑			**OR = 2.37 [CI 1.74, 3.23]**			
						↑		**OR = 2.00 [CI 1.46, 2.81]**			
							↑	**OR = 2.90 [CI 1.97, 4.27]**			
				**Sadness due to lack of family reunification** *(yes vs. no [Ref])*	↑			**OR = 1.41 [CI 1.06, 1.86]**			
						↑		OR = 1.26 [CI 0.93, 1.71]			
							↑	**OR = 1.49 [CI 1.08, 2.05]**			
				**Family conflicts** *(yes vs. no [Ref])*	↑			**OR = 4.87 [CI 2.25, 10.53]**			
						↑		**OR = 2.51 [CI 1.29, 4.92]**			
							↑	**OR = 5.16 [CI 2.56, 10.40]**			
				**Social isolation** *(yes vs. no [Ref])*	↑			**OR = 3.40 [CI 2.39, 4.83]**			
						↑		**OR = 2.42 [CI 1.70, 3.46]**			
							↑	**OR = 3.29 [CI 2.27, 4.78]**			
				**Perceived discrimination** *(yes vs. no [Ref])*	↑			**OR = 5.68 [CI 2.83, 11.41]**			
						↑		**OR = 5.49 [CI 2.79, 10.81]**			
							↑	**OR = 5.96 [CI 2.97, 11.94]**			
				**Financial hardship** *(yes vs. no [Ref])*	↑			**OR = 3.46 [CI 2.14, 5.60]**			
						↑		**OR = 3.46 [CI 2.14, 5.60]**			
							↑	**OR = 4.31 [CI 2.49, 7.45]**			
Walther et al. (2020) [39]	Germany	Diverse refugee sample (*n* = 4,325) 2016	Cross-sectional study	**Language skills**	↓			**β = ‐0.156 [CI -0.261, -0.052]**	PHQ-4	**Geographical factors** (federal states of Germany); **Sociodemographic factors** (sex, age, education, country of origin, marital status); **Post-migration factors** (duration of residence, legal status, seeking family reunification, accommodation, employment, number of (language) courses, social contacts, language skills); **Pre-/Peri-migration-related factors** (number of flight reasons, arrived in host country alone, negative flight experience); **Other factors** (Religiousness)	High
						↓					
							-	-			
				**Language and integration courses attended** (number of courses)	↓			β = ‐0.013 [CI -0.105, 0.079]			
						↓					
							-	-			
				**Seeking family reunification** *(seeking vs. not seeking [Ref])*	↑			**β = 1.111 [CI 0.805, 1.417]**			
						↑					
							-	-			
				**Social contacts** *(time with people from host country)*	↓			**β = -0.176 [CI -0.270, -0.082]**			
						↓					
							-				
				**Social contacts** *(time with people from country of origin)*	↓			β = -0.079 [CI -0.172, 0.013]			
						↓					
							-	-			
				**Legal status** *(subsidiary protection vs. refugee status or asylum [Ref])*	↑			**β = 0.493 [CI 0.021, 0.965]**			
						↑					
							-	-			
				**Legal status** *(awaiting outcome vs. refugee status or asylum [Ref])*	↑			**β = 0.495 [CI 0.288, 0.702]**			
						↑					
							-	-			
				**Legal status** *(suspension of deportation vs. refugee status or asylum [Ref])*	↑			**β = 0.749 [CI 0.137, 1.362]**			
						↑					
							-	-			
				**Employment** *(currently working vs. currently not working [Ref])*	↓			**β = -0.422 [CI -0.710, -0.134]**			
						↓					
							-	-			
				**Employment** *(not seeking work vs. currently not working [Ref])*	↑			β = 0.312 [CI -0.016, 0.640]			
						↑					
							-	-			
				**Accommodation** *(private accommodation vs. refugee housing facility [Ref])*	↓			**β = -0.446 [CI -0.658, -0.233]**			
						↓					
							-	-			

↑ increase, ↓ decrease, *OR* Odds Ratio, *RR* Relative Risk, *β* standardized beta-coefficient, *B* unstandardized beta-coefficient, *CI* Confidence Interval, *N.A.* Not available. *numbers in bold* statistically significant associations, *MMAT* Mixed Methods Appraisal Tool;

Measurements of exposures: CRM-BS Cortes-Rogler-Malgady Bicultural Scale, ESSI ENRICHD Social Support Instrument, LS-S Loneliness Scale-SOEP, MSPSS Multidimensional Scale of Perceived Social Support, PMLD-CL Post-Migration Living Difficulties Checklist, PMLP-CL Post-Migration Living Problems Checklist, RPMS Refugee Post-Migration Stress Scale

Measurements of outcomes: ETI Essen Trauma Inventory, GAD-2 Generalized Anxiety Disorder-2, GAD-7 Generalized Anxiety Disorder-7, HSCL-10 Hopkins Symptoms Checklist-10, HSCL-25 Hopkins Symptoms Checklist-25, HTQ Harvard Trauma Questionnaire, ITQ International Trauma Questionnaire, PCL-5 PTSD Checklist for DSM-5, PDS-5 Posttraumatic Diagnostic Scale for DSM-5, PHQ-2 Patient Health Questionnaire-2, PHQ-4 Patient Health Questionnaire-4, PHQ-9 Patient Health Questionnaire-9, PSS-I PTSD Symptom Scale – Interview Version for DSM-IV

### Layer 1: Individual factors

#### Language skills

Of all studies included, five examined the association between language skills and mental health [32, 34, 36, 38, 39]. Tinghög et al. [38] found that Syrian refugees who reported language difficulties had significantly higher odds of depression (OR = 2.39 [CI 1.78, 3.19]), anxiety disorder (OR = 1.77 [CI 1.30, 2.40]), and PTSD (OR = 2.77 [CI 2.00, 3.83]) than those without difficulties. Solberg et al. [36] applied similar study methods using a sample of asylum seekers with diverse background. While associations for depressive (OR = 1.95 [CI 1.18, 3.23]) and anxiety symptoms (OR = 2.02 [CI 1.26, 3.26]) were comparable to the results of Tinghög et al. [38], refugees with language difficulties showed five times higher odds for PTSD symptoms than those without difficulties (OR = 5.43 [CI 1.87, 5.18]). Schiess-Jokanovic et al. [34] revealed that treatment-seeking Afghan refugees and asylum seekers in the cluster of complex PTSD reported more problems related to language acquisition and barriers but not for PMLD in general. In a German study, lower language skills were not significantly associated with higher odds of depressive symptoms [32]. In contrast, another German study [39] found that an one-unit increase on a 15-point scale measuring language skills is linked to a lower risk of reporting depressive and anxiety symptoms (PHQ-4) (β = -0.156 [CI -0.261, -0.052]). However, no significant association between the attendance of language or integration courses and symptoms of depression or anxiety was observed.

### Layer 2: Social and community networks

#### Family concerns

Overall, four studies examined the association between family concerns and mental health [34, 36, 38, 39]. Based on data representative for refugees in Germany, symptoms of depression and anxiety (PHQ-4) were significantly higher among refugees seeking family reunification compared to those not seeking family reunification (β = 1.111 [CI 0.805, 1.417]) [39]. Tinghög et al. [38] and Solberg et al. [36] found associations between feelings of sadness due to lack of family reunification and symptoms of depression, anxiety, and PTSD. Both studies reported particularly strong associations between family conflicts and mental health problems. Compared to study participants without family conflicts, those who reported family conflicts had almost five (OR = 4.87 [CI 2.25, 10.53]) [38] or nine times (OR = 9.44 [CI 2.81, 31.72]) [36] higher odds for depressive symptoms, more than twice (OR = 2.51 [CI 1.29, 4.92]) [38] or four times (OR = 4.72 [CI 2.09, 10.70]) [36] higher odds for anxiety symptoms, and five (OR = 5.16 [CI 2.56, 10.40]) [38] or almost four times (OR = 3.85 [CI 1.76, 8.42) [36] higher odds for PTSD symptoms.

#### Loneliness and social support

Loneliness and social support were analyzed in ten studies [22, 27, 28, 32, 33, 35–39]. Whenever loneliness or social isolation was studied, it was significantly associated with an increased risk of experiencing symptoms of mental disorders. In a representative German study [32], an one-unit increase on a 12-point scale measuring the degree of loneliness (LS-S) was significantly associated with an 1.143-fold (OR = 1.143 [CI 1.103, 1.184]) higher chance of reporting depressive symptoms. Asylum seekers and refugees who often experienced social isolation in Sweden had more than three times higher odds of showing depressive symptoms (OR = 3.40 [CI 2.39, 4.83] [38]; OR = 3.10 [CI 1.76, 5.44] [36]) as those without isolation. Slightly different associations with the same direction were observed for anxiety and PTSD symptoms [38], although Solberg et al. [36] found fivefold (OR = 5.69 [CI 3.25, 9.96]) higher odds for PTSD symptoms. In contrast, higher ratings on a 25-point social support scale (ESSI) were associated with lower depressive (PHQ-9) (B = -0.32), anxiety (GAD-7) (B = -0.20 [CI -0.39, 0.01]) and PTSD symptoms (PDS-5) (B = -0.55 [CI -0.98, -0.05]) [33]. Applying the same instruments and scales, Gühne et al. [27] found no significant association between social support and symptoms of depression and PTSD. Based on another social support scale (MSPSS), Böge et al. [22] also found no significant association between social support and anxiety symptoms (GAD-7). However, social support was significantly associated with a decreased risk in reporting depressive (β = -0.240) and PTSD symptoms (β = -0.230) [22]. In a Swiss study [28], lower social support was not significantly associated with PTSD symptom severity, but it was associated with higher symptom severity of disturbances of self-organization (DSO) (β = 0.22). Walther et al. [39] showed that higher ratings on a 6-point scale measuring the time spent with Germans was significantly associated with lower risk of experiencing depressive and anxiety symptoms (PHQ-4) (β = -0.176 [CI -0.270, -0.082]), while such an association was not significant for time spent with people from the country of origin. In a prospective cohort study [37], refugees with poor social support showed a significantly higher relative risk for symptoms of depression and anxiety (RR = 6.2 [CI 3.6, 10.8]) compared to those without poor social support. Sengoelge et al. [35] indicated that higher social hardship was associated with higher levels of depression and anxiety (B = 0.786 [CI 0.598, 1.021]), while higher social support was associated with lower symptom levels (B = -0.103). In addition, social support was found to mediate the association between social hardship and mental health problems.

#### Discrimination

Four studies examined the association between perceived discrimination and mental health problems [23, 34, 36, 38]. Tinghög et al. [38] found that refugees who often perceived discrimination showed a more than five times higher chance of depressive (OR = 5.68 [CI 2.83, 11.41]), anxiety (OR = 5.49 [CI 2.79, 10.81]), and PTSD symptoms (OR = 5.96 [CI 2.97, 11.94]) as those without perceived discrimination. Solberg et al. [36] confirmed these associations, but the association for anxiety symptoms was notably smaller (OR = 2.22 [CI 1.01, 4.90]). In another study [23], higher perceived discrimination was significantly associated with higher symptoms of depression (β_t1_ = 0.235 [CI 0.219, 3.552]; β_t2_ = 0.271 [CI 0.383, 2.769]) and anxiety (β_t1_ = 0.263 [CI 0.359, 2.988]; β_t2_ = 0.335 [CI 0.700, 2.738]) at baseline (t1) and follow-up (t2). No significant association was found for PTSD symptoms. Additionally, Schiess-Jokanovic et al. [34] showed no differences in discrimination in their cluster analysis of PTSD symptom patterns.

### Layer 3: Living and working conditions

#### Legal status

More than half of the studies (*n* = 10) examined differences in mental health among asylum seekers and refugees depending on their current legal status [21, 23, 25, 26, 29–32, 37, 39]. Five studies, identified significant associations between an insecure legal status and mental health symptoms [23, 25, 30, 32, 39]. Compared to individuals with refugee or asylum status, Walther et al. [39] found a higher risk of reporting depressive and anxiety symptoms (PHQ-4) for individuals with subsidiary protection status (β = 0.493 [CI 0.021, 0.965]), those awaiting their asylum decision (β = 0.495 [CI 0.288, 0.702]), and those whose deportation was suspended (β = 0.749 [CI 0.137, 1.362]). Another German study [32] confirmed the results showing that refugees with a rejected or undecided asylum application had 1.344-fold higher odds for depressive symptoms (OR = 1.344 [CI 1.062, 1.701]) than those with an accepted asylum application. In addition, the future residence permit validity was significantly associated with symptoms of PTSD, but not with depressive or anxiety symptoms [23, 25]. An one-month increase in future validity of residence permit was negatively associated with PTSD symptoms (ETI) among Syrian refugees in the study by Georgiadou et al. [25] (β = -0.20 [CI -0.58, -0.01]), and in the study by Borho et al. [23] (β = -0.184 [CI -0.388, -0.035]). Another study [30] found differences in reporting anxiety and PTSD symptoms between asylum seekers and individuals with residence permit, but not in depressive symptoms. Five studies found no significant association between legal status and symptoms of depression, anxiety, or PTSD [21, 26, 29, 31, 37].

#### Duration of residence

Six studies examined associations between duration of stay in the host country and mental health status [21, 23, 25, 27, 29, 31]. Nissen et al. [31] found that an one-year increase in duration since arriving in Norway was significantly associated with higher odds of experiencing depressive and anxiety symptoms (OR = 1.52 [CI 1.13, 2.05]; OR = 1.52 [CI 1.14, 2.04]). For PTSD symptoms, such an association missed statistical significance (OR = 1.30 [CI 0.96, 1.75]) [31]. Three studies used multiple linear regression analyses to examine the association between an one-month increase in the duration of residence in Germany and mental health. None of them found significant associations between the duration of residence and depressive (PHQ-9) or PTSD symptoms (ETI, PDS-5) [23, 25, 27]. One study [23] investigated anxiety symptoms (GAD-7) without finding any significant associations. The remaining two studies also found no significant relationship [21, 29].

#### Unemployment and financial hardship

Eleven studies examined the association between unemployment or financial hardship and mental disorders [21, 23, 27, 29, 32, 33, 35–39]. Most of them found strong associations between unemployment or financial hardship and mental health problems. Gühne et al. [27] showed that employed refugees have a lower risk of reporting depressive symptoms (PHQ-9) (β = -2.506) and PTSD symptoms (PDS-5) (β = -4.871) compared to unemployed refugees. In another German study [32], unemployed refugees had 1.483-fold (OR = 1.483 [CI 1.037, 2.121]) higher odds of depressive symptoms than employed refugees. Walther et al. [39] found lower depressive and anxiety symptoms (PHQ-4) (β = -0.422 [CI -0.710, -0.134]) for refugees who reported to have employment compared to those without employment. A study conducted in Italy [21] found no significant association between unemployment and PTSD symptoms. Although there were no statistically significant associations between employment status and depressive (B = -1.51), anxiety (B = -1.70 [CI -3.59, 0.17]), and PTSD symptoms (B = -1.37 [CI -5.40, 2.93]) in a study by Renner et al. [33], an income of less than 500 euros per month was significantly associated with higher PTSD symptoms (PDS-5) (B = 7.04 [CI 0.79, 13.72]). Sengoelge et al. [35] found that financial hardship was positively associated with symptoms of depression and anxiety (HSCL-25) (B = 0.786 [CI 0.598, 1.021]) among asylum seekers living in Sweden. Two other Swedish studies [36, 38] showed that individuals who often experienced financial hardship had about three times higher chances of depressive (OR = 3.58 [CI 1.91, 6.72]; OR = 3.46 [CI 2.14, 5.60]) and anxiety symptoms (OR = 2.95 [CI 1.70, 5.13]; OR = 3.46 [CI 2.14, 5.60]), compared to those without financial hardship. For PTSD, the odds were almost six times (OR = 5.85 [CI 3.14, 10.89]) respectively more than four times (OR = 4.31 [CI 2.49, 7.45]) higher [36, 38]. Finally, Strømme et al. [37] found that refugees with poor economy had a significantly higher relative risk (RR = 4.5 [CI 2.6, 7.9]) for symptoms of depression and anxiety (HSCL-10) compared to those without poor economy.

#### Housing

Five studies analyzed the association between housing and mental health [21, 23, 25, 32, 39]. Results of a German study [39] showed that living in private accommodations was linked to less depressive and anxiety symptoms (PHQ-4) compared to living in refugee housing facilities (β = -0.446 [CI -0.658, -0.233]). Using the same data base, another study [32] found that higher ratings on a 10-point scale measuring housing satisfaction were associated with lower odds of reporting depressive symptoms (OR = 0.943 [CI 0.909, 0.978]). Barbieri et al. [21] conducted a latent class analysis to model symptom profiles of PTSD among treatment-seeking asylum seekers and refugees. For study participants in class 3 (pervasive PTSD), the odds of living in large reception centers with over 1,000 residents was more than 12-times (OR = 12.77 [CI 1.49, 109.44]) higher compared to those in class 2 (high PTSD/threat) and more than six times (OR = 6.68 [CI 1.81, 24.61]) higher compared to those in class 1 (moderate PTSD/avoidance). No significant association was observed for class 2 (high PTSD/threat) compared to class 1 (moderate PTSD/avoidance). Two studies [23, 25] found no significant association between the type of accommodation and symptoms of depression (PHQ-9), anxiety (GAD-7), or PTSD (ETI).

### Layer 4: General socio-economic, cultural and environmental conditions

#### Social status

Two studies analyzed the association between social status and mental health [24, 34]. Costa et al. [24] examined the association between changes in subjective social status regarding the transition from country of origin to host country (Germany) and mental health. Asylum seekers and refugees with a subjective social status mobility of three or more steps downwards showed higher depressive (PHQ-2) (B = 1.048) and anxiety symptoms (GAD-2) (B = 1.006) compared to those with no changes. Furthermore, Schiess-Jokanovic et al. [34] showed no differences in socioeconomical living conditions in PTSD symptom patterns.

#### Acculturation

Only one study investigated the association between acculturation and symptoms of mental disorders [26]. The Dutch study by Groen et al. [26] analyzed the association between acculturation preferences (CRM-BS) and symptoms of depression and anxiety (HSCL-25) and PTSD (HTQ). Results showed that acculturation preferences were not significantly associated with symptoms of depression and anxiety (β = -0.113 [CI -0.625, 0.239]) or PTSD (β = -0.112 [CI -0.642, 0.247]).

#### Accumulated postmigration living difficulties

Three studies examined the association between accumulated PMLD, as measured by the PMLD checklist or adapted versions, and mental health [26, 28, 29]. In a Swiss study [28], more PMLD were significantly associated with higher DSO symptom severity (β = 0.42). Groen et al. [26] showed that PMLD were positively associated with symptoms of anxiety and depression (HSCL-25) (β = 0.428 [CI 0.170, 0.710]) and PTSD symptoms (HTQ) (β = 0.396 [CI 0.140, 0.713]) among asylum seekers and refugees living in the Netherlands. In a German study [29], the experience of less PMLD in the past year was associated with fewer PTSD symptoms during the year (estimate = -6.97 (2.77), [-12.49, -1.45]). No significant associations were found for depressive symptoms (estimate = -2.19 (1.34), [-4.86, 0.47]).

## Discussion

The aim of this review was to examine and narratively describe the associations between characteristics of the postmigration living situation (PMLS) and mental health outcomes among asylum-seekers and refugees who lived in European countries between 2015 and 2022. We used the social determinant of health framework by Dahlgren and Whitehead [12] to cluster the characteristics of the PMLS according to four layers, yielding the following core findings:

1. Individual factors, in particular low language skills, were frequently associated with unfavorable mental health outcomes, whereas obtaining language skills were associated with less symptoms.
2. Weak social and community networks including loneliness were consistently associated with unfavorable mental health outcomes, as was family concerns and discrimination in most studies. In contrast, high social support frequently appeared to be associated with lower symptoms of mental disorders.
3. Among living and working conditions, legal status was most commonly studied; insecure legal status was a strong risk factor in some studies, although half of the studies found no significant associations. Independently, unemployment, financial hardship, and being housed in (large) facilities showed strong associations with symptoms of mental disorders.
4. General socio-economic, cultural, and environmental factors including social status and acculturation showed inconsistent or weak associations with unfavorable mental health outcomes, unless they were part of summary measures of PMLD.

Thus, an important new insight of our review is that when broken down in its component layers, characteristics of the PMLS were significantly associated with symptoms of mental disorders showing the same direction of association across the included studies, while the association between some stressors or resources of the PMLS and mental health turn out to be less homogeneous than expected. Some patterns are discernible: family conflicts tend to be strongly associated with symptoms of mental disorder, and social isolation consistently does so; discrimination tends to be associated with symptoms of mental disorders. Our review highlights the importance for political action to reduce inequalities and strengthen mental health in refugee and asylum seekers. Many of the identified social determinants can be influenced by policy changes, such as access to the housing and labor market, simplified legal conditions for asylum procedures and family reunification, or low-threshold offers for language acquisition.

In addition, our review highlights the need for high quality longitudinal studies to fully understand the living situation of refugees in host countries. In order to measure long-term mental health effects, it is indispensable to consider changes in the living situation, for example through the acquisition of a long-term residence permit or access to work and education. Therefore, it is necessary that both stressors and resources are taken into account.

Differences in findings between studies can be partly explained by the measurement of expositions. For assessing language skills, for example, the Swedish studies [36, 38] asked about language difficulties, whereas the German studies asked about linguistic competencies [32, 39]. One problem in all studies was that language skills were self-rated and no standardized assessment was used. The same applies to social support, where Tinghög et al. [38] and Solberg et al. [36] calculated high odds ratios using single items, whereas Gühne et al. [27] found no associations using standardized assessments.

Statistical analysis may influence results: For example, Walther et al. [39] formed a mean index from three Likert scales for writing, reading and speaking German finding associations between German language ability and mental health in their linear regression model, while Nutsch & Bozorgmehr [32] used a dichotomized variable in their logistic regression analysis finding no associations in the same population. We saw similar challenges with other PMLD, such as social support: Two studies that assessed social isolation [36, 38] found a strong association with poor mental health. In comparison, one study that used a standardized assessment observed no associations between social support and mental health when comparing employed and unemployed refugees.

Furthermore, living conditions in host countries may be of different importance for mental health. Four of five studies conducted in Germany found significant associations between legal status and depressive and anxiety symptoms [32, 39] and PTSD [23, 25]. In comparison, Kaltenbach et al. [29] found no associations between legal status and PTSD and depression in a longitudinal study, but the sample size was small (n = 57) and most participants just started their asylum procedure (n = 40). All other included studies found no associations. Because results regarding the association between legal status and mental health are heterogenous, further studies [40, 41] indicated that changes in living situation and possibilities for social participation are much more decisive than legal status.

Evidence is scarce regarding the influence of acculturation on mental health. This is possibly due to different definitions and operationalizations [42]. Moreover, the concept is not free of criticism [43]. Only one study investigated the role of acculturation among refugees in Europe but showed no significant associations with mental health [26]. Thus, a clearer understanding of the concept of acculturation and its relevance to mental health in refugee populations is necessary.

Our review partly confirms the results of a meta-analysis by Hou et al. [7] and a systematic review by Gleeson et al. [44], which showed that PMLD were negatively associated with mental health outcomes. However, similar to our narrative analysis, the authors concluded that the influence of PMLD on psychiatric disorders differ. Their meta-analysis showed that subjective daily stressors, were associated with anxiety and PTSD but not with depressive symptoms, and that material daily stressors were associated with PTSD only. Our narrative review, which is based on current literature, also highlights the lack of comparability of study results due to differences in the measurement of characteristics of the PMLS, heterogeneity of study populations and different reception conditions. By using the framework on social determinants of health, we wanted to achieve a broader understanding of the general living situation of refugees and asylum seekers in Europe, in which not only stressors but also potential protective factors (e.g. language skill, social status, social support) could be identified. A comprehensive understanding of the living situation of refugees and asylum seekers through the social determinants of health lens can also be helpful in understanding the living situation and health outcomes of other marginalised groups, for example homeless persons, thus helping to improve health and social care [45, 46].

In summary, PMLD can partly contribute to the high prevalence of psychiatric disorders in refugee and asylum-seeking populations. However, due to the study designs no assumptions about causal relationships can be drawn. According to Schick et al. [47] it can be assumed that mental disorders may also be a barrier to integration. Further studies thus need to gain a deeper understanding of the role of mental health on perceived resources in host countries, such as types of social support, acculturation, accommodation and language skills.

### Strengths and limitations

A strength of this review is the systematic approach according to the PRISMA guidelines. By incorporating recently published literature and clustering characteristics of the PMLS within the model of social determinants of health, the complexity of the PMLS was made clear. Nevertheless, there are some limitations that need to be considered. Most studies examined heterogeneous study populations, with the possible consequence that flight and traumatic experiences in countries of refuge are difficult to compare. Characteristics of the PMLS were not always systematically recorded. This makes it difficult to compare results. The heterogeneity of the primary studies did not allow for meta-analysis of the extracted data. Additionally, the political-legal situation in the European host countries as well as the status of different migrant populations differ although there is legal guidance at the EU level. The transposition into national law and the local social situation can lead to different forms of inclusion and exclusion mechanisms. This may lead to differences in the (subjective) relevance of specific stressors or resources in the PMLS. In this regard, the comparability of findings about associations between characteristics of the PMLS and mental health status is limited. It is possible that the inclusion of non-European literature and consideration of populations with a longer duration of stay in Europe would have contributed to further valuable findings.

## Conclusions

Our systematic review provides an up-to-date overview of the associations between characteristics of the PMLS and mental health among refugees who lived in Europe from 2015 onwards. Different forms of disadvantage among refugees and asylum seekers in host countries became apparent when placed within the model of social determinants of health. Broken down in its component layers, most risk factors and protective factors of the PMLS were significantly associated with mental health problems, showing the same direction of association. Only the general socio-economic, cultural and environmental conditions showed weak and unclear associations with mental diseases. Our updating systematic review thus follows up on previous published systematic reviews. It highlights again the importance of the postmigration living situation for mental health among refugees and asylum seekers, showing that it continues to apply also to those who lived in Europe from 2015 onwards. Our review contributes to the growing body of evidence of the effects of postmigration living difficulties and resources and mental health. The lessons learned so far are also reflected in this review. The diverse stressors and resources resulting from the social determinants of health framework are like a magnifying glass for different forms of advantages and disadvantages that can affect mental health also of other marginalized populations in European countries, such as homeless persons or migrant workers in precarious jobs.

## Supplementary Information

**Additional file 1.**

## Data Availability

All data generated or analyzed during this study are included in this published article.
