# Supplementary material for: Associations between postmigration living situation and symptoms of common mental disorders in adult refugees in Europe: updating systematic review from 2015 onwards
Source: BMC Public Health. 2023 Jul 5;23:1289. doi: 10.1186/s12889-023-15931-1 (PMC10320886; doi:10.1186/s12889-023-15931-1)
Supplement: Supplementary file 1 — Additional file 1. [file 12889_2023_15931_MOESM1_ESM.docx]

**Additional File 1: Search strategies in the databases**

1. **Pubmed (28.02.2022)**

| ID | Query | Results |
| --- | --- | --- |
| #21 | **#19 AND #20** | 2,098 |
| #20 | **(2015/01/01:2022/02/22[edat])** |  |
| #19 | *#3 AND #12 AND #18* |  |
| #18 | *#13 OR #14 OR #15 OR #16 OR #17* |  |
| #17 | posttraumatic stress disorder OR ptsd |  |
| #16 | anxiety OR (anxiety AND (disorder OR symptoms)) |  |
| #15 | depression OR (depressive AND (disorder OR symptoms)) |  |
| #14 | (mental OR psychological OR psychiatric) AND (health OR disorder OR illness) |  |
| #13 | "Mental Health"[Mesh] OR "Mental Disorders"[Mesh] |  |
| #12 | *#4 OR #5 OR #6 OR #7 OR #8 OR #9 OR #10 OR #11* |  |
| #11 | discrimin* OR racism |  |
| #10 | loneliness OR lonely OR "family separation" OR "community integration" OR (social AND (isolat* OR exclusion OR support OR networks OR relationships OR inclusion OR participation OR integration)) |  |
| #9 | ((language OR communication) AND (acquisition OR skills OR barriers OR problems OR proficiency)) |  |
| #8 | employ* OR unemploy* |  |
| #7 | accommod* OR housing |  |
| #6 | "asylum proce*" OR ((legal OR resident OR residency OR residence) AND (status OR permit)) |  |
| #5 | ((postmigration OR "post migration" OR "post-migration") AND ("living difficulties" OR difficulties OR stress OR stressors OR factors)) OR pmld |  |
| #4 | "Housing"[Mesh] OR "Employment"[Mesh] OR "Unemployment"[Mesh] OR "Income"[Mesh] OR "Communication Barriers"[Mesh] OR "Loneliness"[Mesh] OR "Social Isolation"[Mesh] OR "Social Support"[Mesh] OR "Social Inclusion"[Mesh] OR "Social Integration"[Mesh] OR "Social Discrimination"[Mesh] OR "Racism"[Mesh] |  |
| #3 | *#1 OR #2* |  |
| #2 | refugee* OR (asylum AND (seek* OR applicants)) OR (displaced AND (persons OR people)) OR (forced AND (migrants OR migration OR displacement)) |  |
| #1 | "Refugees"[Mesh] |  |

1. **Web of Science (28.02.22)**

| **ID** | **Query** | **Results** |
| --- | --- | --- |
| #17 | **#15 AND #16** | **2,477** |
| #16 | **DOP=(2015-01-01/2022-02-28)** |  |
| #15 | *#1 AND #9 AND #14* |  |
| #14 | *#10 OR #11 OR #12 OR #13* |  |
| #13 | TS = "posttraumatic stress disorder" OR ptsd |  |
| #12 | TS = anxiety OR (anxiety AND (disorder OR symptoms)) |  |
| #11 | TS = depression OR (depressive AND (disorder OR symptoms)) |  |
| #10 | TS = (mental OR psychological OR psychiatric) AND (health OR disorder OR illness) |  |
| *#9* | *#2 OR #3 OR #4 OR #5 OR #6 OR #7 OR #8* |  |
| #8 | TS = discrimin* OR racism |  |
| #7 | TS = loneliness OR lonely OR "family separation" OR "community integration" OR (social AND (isolat* OR exclusion OR support OR networks OR relationships OR inclusion OR participation OR integration)) |  |
| #6 | TS = ((language OR communication) AND (acquisition OR skills OR barriers OR problems OR proficiency)) |  |
| #5 | TS = employ* OR unemploy* OR income |  |
| #4 | TS = accommod* OR housing |  |
| #3 | TS = "asylum proce*" OR ((legal OR resident OR residency OR residence) AND (status OR permit)) |  |
| #2 | TS = ((postmigration OR "post migration") AND ("living difficulties" OR difficulties OR stress OR stressors OR factors)) OR pmld |  |
| #1 | TS = refugee* OR (asylum AND (seek* OR applicants)) OR (displaced AND (persons OR people)) OR (forced AND (migrants OR migration OR displacement)) |  |

1. **PsycINFO und PSYNDEX via EBSCO Host (28.02.2022)**

| **ID** | **Query** | **Results** |
| --- | --- | --- |
| #17 | **#15 AND #16** | **1,809** |
| #16 | **Limiters: 20150101-20220228** |  |
| #15 | *#1 AND #9 AND #14* |  |
| #14 | *#10 OR #11 OR #12 OR #13* |  |
| #13 | TX ("posttraumatic stress disorder" OR ptsd) |  |
| #12 | TX (anxiety OR (anxiety AND (disorder OR symptoms))) |  |
| #11 | TX (depression OR (depressive AND (disorder OR symptoms))) |  |
| #10 | TX ((mental OR psychological OR psychiatric) AND (health OR disorder OR illness)) |  |
| #9 | *#2 OR #3 OR #4 OR #5 OR #6 OR #7 OR #8* |  |
| #8 | TX (discrimin* OR racism) |  |
| #7 | TX (loneliness OR lonely OR "family separation" OR "community integration" OR (social AND (isolat* OR exclusion OR support OR networks OR relationships OR inclusion OR participation OR integration))) |  |
| #6 | TX ((language OR communication) AND (acquisition OR skills OR barriers OR problems OR proficiency)) |  |
| #5 | TX (employ* OR unemploy* OR income) |  |
| #4 | TX (accommod* OR housing) |  |
| #3 | TX ("asylum proce*" OR ((legal OR resident OR residency OR residence) AND (status OR permit))) |  |
| #2 | TX ((postmigration OR "post migration") AND ("living difficulties" OR difficulties OR stress OR stressors OR factors)) OR pmld |  |
| #1 | TX (refugee* OR (asylum AND (seek* OR applicants)) OR (displaced AND (persons OR people)) OR (forced AND (migrants OR migration OR displacement))) |  |

1. **Google Scholar**

| **ID** | **Query** | **Results** |
| --- | --- | --- |
| #4 | #1 AND #2 AND #3 | **427** |
| #3 | Mental health |  |
| #2 | “postmigration stress” |  |
| #1 | (refugees OR asylum seekers) |  |

**Time Frame: 2015-2022**
